# An evaluation of lipid profile and pro-inflammatory cytokines as determinants of cardiovascular disease in those with diabetes: a study on a Mexican American cohort

**DOI:** 10.1038/s41598-021-81730-6

**Published:** 2021-01-28

**Authors:** Amna Tahir, Perla J. Martinez, Fayyaz Ahmad, Susan P. Fisher-Hoch, Joseph McCormick, Jennifer L. Gay, Shaper Mirza, Safee Ullah Chaudhary

**Affiliations:** 1 Biomedical Informatics Research Laboratory, Department of Biology, Lahore University of Management Sciences, Lahore, Pakistan; 2 Department of Epidemiology, Human Genetics and Environmental Health, School of Public Health, University of Texas Health Science Center – Brownsville Regional Campus, Brownsville, USA; 3 Department of Statistics, University of Gujrat, Gujrat, Pakistan; 4 Department of Health Promotion and Behavior, College of Public Health, University of Georgia, Athens, USA; 5 Microbiology and Immunology Laboratory, Department of Biology, Lahore University of Management Sciences, Lahore, Pakistan

**Keywords:** Biomarkers, Cardiology, Diseases, Health care, Medical research, Risk factors

## Abstract

Sedentary life styles coupled with high-calorie diets and unhealthy social habits such as smoking, have put an ever-increasing number of people at risk of cardiovascular disorders (CVD), worldwide. A concomitant increase in the prevalence of type 2-diabetes (hyperglycemia), a risk factor for CVD, has further contributed towards escalating CVD-related mortalities. The increase in number of cases of type 2-diabetes underscores the importance of early diagnosis of cardiovascular disease in those with diabetes. In this work, we have evaluated the sensitivity and specificity of dyslipidemia and proinflammatory cytokines to be used as biomarkers for predicting the risk of CVD in those with diabetes. We hypothesize that interplay between dyslipidemia and diabetes-induced low-grade inflammation in those with type 2-diabetes increases the risk of CVD. A total of 215 participants were randomly recruited from the Cameron County Hispanic Cohort (CCHC). Of these, 99% were Mexican Americans living on Texas-Mexico border. Levels of cytokines, adipokines and lipid profile were measured. Cardiovascular disease (CVD) for this study was defined as prior diagnosis of heart attack, angina and stroke, while diabetes was defined by fasting blood glucose (FBG) of > 100 mg/dL and HbA1c of > 6.5, in accordance with American Diabetes Association (ADA) guidelines. Depending on type and distribution of data, various statistical tests were performed. Our results demonstrated higher rates of heart attack (14% vs 11.8%) and stroke (19.8% vs 10%) in those with diabetes as compared to non-diabetes. The odds of having a heart attack were eight times higher in the presence of elevated triglycerides and pro-inflammatory markers (TNFα and IL6) as compared to presence of pro-inflammatory markers only. The odds for heart attack among those with diabetes, increased by 20 fold in presence of high levels of triglycerides, TNFα, and IL6 when coupled with low levels of high-density lipid cholesterol (HDL-C). Lastly, our analysis showed that poorly controlled diabetes, characterized by HbA1c values of > 6.5 increases the odds of stroke by more than three fold. The study quantifies the role of lipid profile and pro-inflammatory markers in combination with standard risk factors towards predicting the risk of CVD in those with type 2-diabetes. The findings from the study can be directly translated for use in early diagnosis of heart disease and guiding interventions leading to a reduction in CVD-associated mortality in those with type 2-diabetes.

## Background

Sedentary and unhealthy lifestyles have put an ever-increasing number of people at the risk of developing cardiovascular disorders (CVD)^1^, which is indicated by an alarming rise in incidence of heart failure, cardiomyopathy, coronary heart disease, and stroke^2^. American National Health and Nutrition Examination Survey (NHANES) shows that incidence of heart failure (HF) increased by about 9% (from 5.7 million to 6.2 million) in adults ≥ 20 years of age (2.2% of population) from 2013–2016 to 2009–2012^3^. The Global Burden of Disease Study reports that approximately 90% of stroke incidents can be attributed to modifiable risk factors emerging from sedentary and unhealthy life styles. Specifically, smoking, high body mass index (BMI), sub-optimal physical activity coupled with high calorie diets, high cholesterol, elevated blood pressure (BP), diabetes (hyperglycemia) and renal dysfunction have been reported as key determinants of cardio vascular disease (CVD)^3,4^. Furthermore, the surge in prevalence of type 2-diabetes (hereafter referred to as diabetes) has significantly contributed to escalation in CVD-related mortalities. Hence, diabetes with uncontrolled fasting glucose ≥ 6.5 mmol/L^1,5^ has also emerged as an important independent risk factor for cardiovascular disease (CVD)^6–9^. In 2007, citing the axis between diabetes and CVD, American Heart Association (AHA)and American Diabetes Association (ADA) issued a combined set of recommendations focusing on prevention of CVD in those with diabetes^10^.

Diabetes was identified as a major risk factor for CVD after conducting a 20 year surveillance study on Framingham cohort aimed at identifying risk factors for cardiovascular disease^11^. Krauss et al*.* evaluated the association of type 2 diabetes with lipid and lipoprotein abnormalities^12^ while Athyros et al*.* highlighted the high prevalence of dyslipidemia (DD)^13^ amongst the patients with type 2 diabetes. Cannon et al*.* reported that metabolic syndrome superimposed on type 2 diabetes augments the risk of CVD^14^ while Cao et al*.* stressed upon the increased risk of CVD as a result of elevated non-HDL-C levels amongst type 2 diabetes population^15^. Recently, Pagidipati et al.further reported hypertriglyceridemia’s involvement in giving rise to CVD related mortalities in individuals with diabetes^16–19^. In concert, numerous research reports have classified lipid profile and blood pressure amongst major risk-factors that exhibit a stronger association with CVD than poorly controlled glucose in those with diabetes^20^. Besides the lipid profile, Tuttle et al.have shown that increased levels of the TNFα and IL6 in diabetes is also associated with elevated morbidity and mortality rates in CVD^21^. Inflammatory markers such as IL6, TNFα, C-reactive protein (CRP) and IL8-β are well-established mediators in the induction of cardiovascular diseases^22–28^. Ridker et al*.* demonstrated that C-reactive protein (CRP) (an inflammatory marker in the blood) and lipid profile (total cholesterol to HDLC) increased the relative risk of cardiovascular events in women^29^. Epidemiological studies have also reported associations between inflammatory markers and type 2 diabetes through elevated plasma concentration of inflammatory mediators, such as tumor necrosis factor-α (TNF-α) and interleukin-6 (IL-6)^30–32^. Summarily, meta-analysis of literature does not demonstrate that diabetes is a CVD risk equivalent^33,34^ and that there is a lack of consensus on which individual risk factors^35,36^ or their combinations^37,38^ are the most crucial in giving rise to CVD amongst those with a history of type 2 diabetes. In particular, the interplay of type 2 diabetes-induced low-grade inflammation with lipid abnormalities in the risk of CVD, remains poorly understood.

To address this gap, we aim to estimate the association of lipid profiles and inflammatory markers with the risk of developing CVD, in Mexican Americans. Next, we investigate the combinatorial utility of these modifiable risk factors in predicting the risk of CVD in those with type 2-diabetes, by clustering lipid profiles and inflammatory markers. Lastly, we validate the effect of superimposition of diabetes-induced inflammatory cytokines on lipid profile on increasing the odds of developing CVD. Our results show that lipid profile and inflammatory cytokines are predictive biomarkers of cardiovascular disease especially in those with diabetes, which can be employed in clinical settings towards early diagnosis of heart disease. We further report that the odds of having heart attack in presence of elevated triglycerides and pro-inflammatory parameters (TNFα and IL6) were approximately eight times higher as compared to heart attack in individuals with only elevated levels of pro-inflammatory markers. However, the odds of having a heart attack in participants with diabetes increased by 20 fold in presence of high levels of triglycerides, TNFα and IL6 if coupled with low levels of HDL-C. Lastly, for stroke, our analysis showed that uncontrolled diabetes increases its odds by over three times.

## Methods

### Study participants and demographics

We measured level of cytokines and adipokines in 215 participants from the Cameron County Hispanic Cohort (CCHC). The Cameron County Hispanic Cohort (CCHC) was formed in 2004 and comprised of Mexican Americans inhabiting the United States-Mexico border^39–42^. Previous studies performed on the border communities have indicated that Latinos have 1.4 times higher risk of diabetes than those reported nationally for Mexican Americans^43^. Therefore, CCHC randomly recruited 2000 residents from City of Brownsville for onward investigations. Since earlier studies on CCHC reported higher levels of inflammatory markers (TNF-alpha and IL6) amongst Mexican Americans participants with diabetes^32^, the current study was designed to further investigate the crosstalk between inflammation, diabetes and the risk of CVD.

The American Diabetes Association goal for glycemic control of HbA1c < 6.5% in the Standards of Medical Care in Diabetes-American Diabetes Association, Diabetes Care 2010, Supplement 1-S11^1^ was used to identify diabetes. Moreover, each participant had a clinical diagnosis of diabetes or impaired fasting blood glucose for at least 2 years prior to the start of the study while some of them were also on medication for diabetes. None of the participants had any hemoglobinopathies nor had undergone blood transfusion. The selected participants were selected on the following criteria, (i) diabetes no cardiovascular disease, (ii) diabetes with cardiovascular disease, (iii) no diabetes and no cardiovascular disease. The study was approved by the Institutional Review Board of University of Texas, Health Science Center, School of Public Health and all methods were performed in accordance with the relevant guidelines and regulations. The study was explained to participants in both English and Spanish and informed consent was obtained. After signing of the consent form, participants were requested for 10mls of blood for onward cytokine analysis as well as for quantitation of metabolic markers. Demographic and anthropometric data were collected using a questionnaire.

### Study population and selection criteria

Self-report of a previous clinical diagnosis or a random blood glucose test were used to determine the diabetes status of participants in the study. The criteria defined by the American Diabetes Association (ADA) in their “standards of medical care in diabetes—2010”^1^ was used to classify diabetes. Specifically, participants were said to have type 2-diabetes if they fulfilled any one of the following criteria; Fasting blood glucose (FBG) > 100 mg/dl and HbA1c > 6.5; a doctor’s diagnosis of diabetes and/or on medication for diabetes^44^. Absence of diabetes was defined as no history of diabetes and FBG < 100 mg/dl. Diabetes control over three month period was measured by using glycated hemoglobin (HbA1c), where a value of < 6.5 was considered well-controlled diabetes, whereas HbA1c > 6.5 indicated poor glucose control^44^. Cardiovascular disease was also self-reported based on history of congestive heart failure, coronary heart disease, angina, myocardial infarction or hypertension. Three sub-cohorts were henceforth defined with (i) participants having type 2-diabetes with any of the following cardiovascular issues: hypertension, angina, heart attack, and high cholesterol, (ii) cardiovascular disease but no diabetes, and (iii) no cardiovascular disease and no diabetes.

### Measurement of analytes in serum samples

Blood was kept at room temperature until it was transported to the laboratory at the School of Public Health University of Texas, Brownsville Campus. Blood was allowed to clot followed by centrifugation and separation of serum. Serum was divided in 500 µl aliquots and kept frozen at − 80 °C. Levels of cytokines and adipokines, were measured using a multiplex ELISA technology (Milliplex Map, Millipore CA), followed by reading of results in Luminex 200 system (Luminex corp. Austin TX). Measurement of cytokines and adipokines was made using two panels, wherein panel-A comprised of adiponectin and resistin, panel-B contained probes for pro-inflammatory cytokines IL-6, TNF-alpha, IL-1beta, and leptin. Plasma samples stored at − 80 °C were thawed on ice, diluted as per manufacturer’s instructions, and incubated with beads coated with antibodies for corresponding analytes. Reaction was read in the Luminex 200 system.

### Statistical analysis

International Business Machine (IBM)—Statistical Package for Social Sciences (SPSS) version 23^45^ and Stata/MP version 13 were used for data analysis^46^. Data was evaluated for normality using Kolmogorov–Smirnov^47^ and Shapiro–Wilk tests^48^. In accordance with the distribution of independent variables, non-parametric methods including Mann Whitney U test and parametric tests including independent two-sample t-test were employed for analysis of data. Anthropometric measures, lipid profile, adipokines and inflammatory markers were compared by diabetes status, (HbA1c) status, CVD (heart attack, stroke and angina) status by stratification of the cohort using Mann Whitney U test^49^. Prevalence of CVD was assessed with 95% confidence intervals (CI) amongst those with and without diabetes. Relative risk, after adjusting for age and BMI, was computed for CVD in those with diabetes. Chi-square^50^ and Cochran–Mantel–Haenszel^51^ tests were used to calculate odds of CVD as well as the association of lipid profile and inflammatory markers with CVD in those with and without diabetes. Multilayer Perceptron neural network^52^ was trained to test for CVD risk factors. Analyses were declared significant for P-value < 0.05.

## Results

### Evaluation of baseline anthropometric and metabolic characteristics in those with and without diabetes

Participants with diabetes mellitus (DM) and without diabetes (nDM) were evaluated for anthropometric and metabolic characteristics (Table 1). From amongst the cohort, a sample of 91 participants were self-reported to have had one or more of the following; angina, heart attack or stroke which were collectively termed as “cardiovascular diseases”. Out of 215 participants, 44.7% had diabetes of which 66.7% were females and 33.3% were males while for those without diabetes (55.4% of 215 participants), 58% were female and 42% were male. The ratio between genders in those with and without diabetes was found to be comparable by comparison of frequency of valid (non-missing) observations in both DM and nDM groups. Next, we compared the DM and nDM groups across 14 different factors. These included age, body mass index (BMI), mean fasting blood glucose (FBG), glycated hemoglobin (GHB), triglycerides, high-density lipoprotein (HDL), leptin, systolic and diastolic blood pressure, receptor for advanced glycation end (RAGE), C-reactive protein (CRP), cholesterol, resistin and adiponectin. Non-parametric Mann–Whitney U test reported no significant difference between both genders amongst those with and without diabetes (P < 0.05). Age and BMI were significantly higher in diabetes as compared to non-diabetes (P = 0.0014, and P = 0.0007), respectively. A significant increase was observed in levels of HbA1c (5.5 vs. 7.4%), mean FBG (94 vs. 127.5 mg/dl), triglycerides (169.5 vs. 152 mg/dl, P = 0.0007), and systolic blood pressure (125.5 vs. 119 mm/Hg, P = 0.0003) in diabetes. However, a low HDL-C (45 vs. 48 mg/dl P = 0.01) was observed in DM as compared to nDM. Further, no significant difference was observed in adipokines (including leptin, adiponectin and resistin), in participants with and without diabetes.

**Table 1:** Evaluation of baseline characteristics by diabetes status.

Baseline characteristics	nDM (n = 119)	DM (n = 96)	Test statistics Mann–Whitney U	P-value
**Gender**				
Male	42%	33.30%	5216	0.19
Female	58%	66.67%		
**Age (years)**	58	63.5	4187.5	0.0014**
**BMI (Kg/m**^**2**^**)**	30.3	31.3	4127.5	0.001**
**Mean FBG (mg/dL)**	94	127.5	1839	0.0000 **
**% HbA1c**	5.5	7.4	899	0.0000 **
**Triglycerides (mg/dL)**	152	169.5	4105.5	0.0007**
**IL-6 (pg/ml)**	2.5	4.1	4518.5	0.007**
**TNFa (pg/ml)**	5.0	5.4	4258.5	0.0013**
**HDL-C (mg/dL)**	48	45	4466	0.01*
**Systolic BP (mm/Hg)**	119	125.5	4069	0.0003**
Leptin (ng/ml)	22,402.7	25,595.1	5086	0.167
Adiponectin (ug/ml)	16.6	17.12	5606	0.815
IL-8 (pg/ml)	3.6	3.5	4838	0.051
Resistin (ng/ml)	1.3	1.1	5327.5	0.114
RAGE (pg/ml)	956	1022.6	2706	0.307
Cholesterol (mg/dL)	189	179	5216	0.384
CRP (mg/L)	0.4	0.5	5166	0.28
Diastolic BP (mm/Hg)	72	66	4889.5	0.07

Median values for baseline anthropometric and metabolic characteristics of participants, compared by diabetes (DM) and non-diabetes (nDM) status. The significant parameters and their respective p-values are highlighted in bold.

*P < 0.05, **P < 0.01.

### Measurement of pro and anti-inflammatory markers by diabetes status in absence of CVD

To evaluate the inflammatory response in diabetes, we compared cytokine expressions between those with and without diabetes using Mann–Whitney U test. The cytokines evaluated included both pro-inflammatory (IL-6, TNFα, and IL1β) and anti-inflammatory (IL8) markers. Our results show that DM in comparison to nDM, exhibits a significant increase in levels of pro-inflammatory IL-6 (4.1 vs 2.45, P = 0.007) and TNFα (5.385 vs 5.03, P = 0.0013) (Table 1). To understand the role of glucose control on inflammation we compared levels of cytokines among participants with HbA1c < 6.5% (controlled) and > 6.5 (poorly controlled). Non-parametric Mann–Whitney U test was used for these comparisons, however, no significant differences were found within cytokines and adipokines (data not shown).

### Comparison of baseline anthropometric, inflammatory and metabolic characteristics by diabetes status in absence of cardiovascular disorders

To further analyze baseline parameters in participants without any cardiovascular disease (CVD), we stratified the cohort by CVD status (n = 123). The Mann–Whitney U test was employed and results are shown in Table 2. The results obtained were consistent with our previous findings (Table 1) except for HDL-C and triglycerides, which were not significant in those with diabetes and no CVD. This analysis helped identify factors that might be contributing significantly towards cardiovascular in the presence of diabetes.

**Table 2:** Evaluation of baseline characteristics by diabetes status with no CVD.

Baseline characteristics	nDM (n = 75)	DM (n = 48)	Test statistic Mann–Whitney U	P-value
**Gender**				
Male	40%	31.30%	1702.5	0.41
Female	75%	68.80%		
**Age (years)**	55	63.5	1180	0.0006***
**BMI**	30.1	32.3	1281	0.0083**
**Mean FBG (mg/dL)**	95	118.5	678.5	0.0000***
**% HbA1c**	5.4	7.4	335	0.0000***
**Systolic BP (mm/Hg)**	116	125.5	1259	0.0022**
**IL6 (pg/ml)**	0.7	2.9	1384.5	0.0152*
**TNFα (pg/ml)**	4.9	5.5	1390	0.0200*
Leptin (ng/ml)	20,365.1	23,764.6	1541	0.0871
Adiponectin (ug/ml)	15.12	17.1	1645.5	0.2361
IL-8 (pg/ml)	3.07	3.33	1561.5	0.150
RAGE (pg/ml)	952.8	1068.8	654	0.1273
Cholesterol (mg/dL)	181.5	188	1609.5	0.4843
Triglycerides (mg/dL)	122.5	137	1483.5	0.1118
HDL-C (mg/dL)	47.5	46	1521.5	0.2364
LDLCALC (mg/dL)	113	110	1391	0.2475
CRP (mg/L)	0.4	0.5	1656	0.3348
Diastolic BP (mm/Hg)	70	67	1593.5	0.192

Median values for baseline anthropometric, metabolic and inflammatory characteristics of participants, compared by diabetes (DM) and non-diabetes (nDM) status with no episode of cardiovascular disorder. The significant parameters and their respective p-values are highlighted in bold.

*P < 0.05, **P < 0.01.

### Evaluation of baseline anthropometric and metabolic characteristics in participants with cardiovascular diseases with and without diabetes

Next, we set out to analyze the full subset (n = 215) for cardiovascular diseases regardless of diabetes status. Specifically, anthropometric, metabolic characteristics and pro-inflammatory characteristics were evaluated by heart attack and stroke status. Mann Whitney U test was performed and our results (Table 3-A) indicate that participants with a history of a heart attack showed a significant increase in the levels of triglycerides (226.5 mg/dL) as compared to patients without any history of heart attack (138 mg/dL). Glycated hemoglobin (GHB) showed a significant increase from 5.8% to 7% in individuals with history of stroke as compared to those who never had a stroke (Table 3-B).

**Table 3:** Evaluation of characteristics by CVD status.

A. Characteristics by heart attack status				
Lipid profile	No heart attack (n = 184)	Heart attack (n = 28)	Test statistic Mann–Whitney U	P-value
Triglycerides (mg/dL)	138	226.5	1389	0.000087**

B. Characteristics by Stroke status				
Glucose control measure	No stroke (n = 184)	Stroke (n = 31)	Test statistic Mann–Whitney U	P-value
GHB (glycated hemoglobin)	5.8	7	2061.5	0.0135*

Median values for different characteristics of participants compared by (A) heart attack, and (B) stroke status. Only the significant variables are shown above with their respective p-values highlighted in bold.

*P < 0.05, **P < 0.01.

### Prevalence estimates of CVD and its risk assessment in those with diabetes

**Figure 1:**
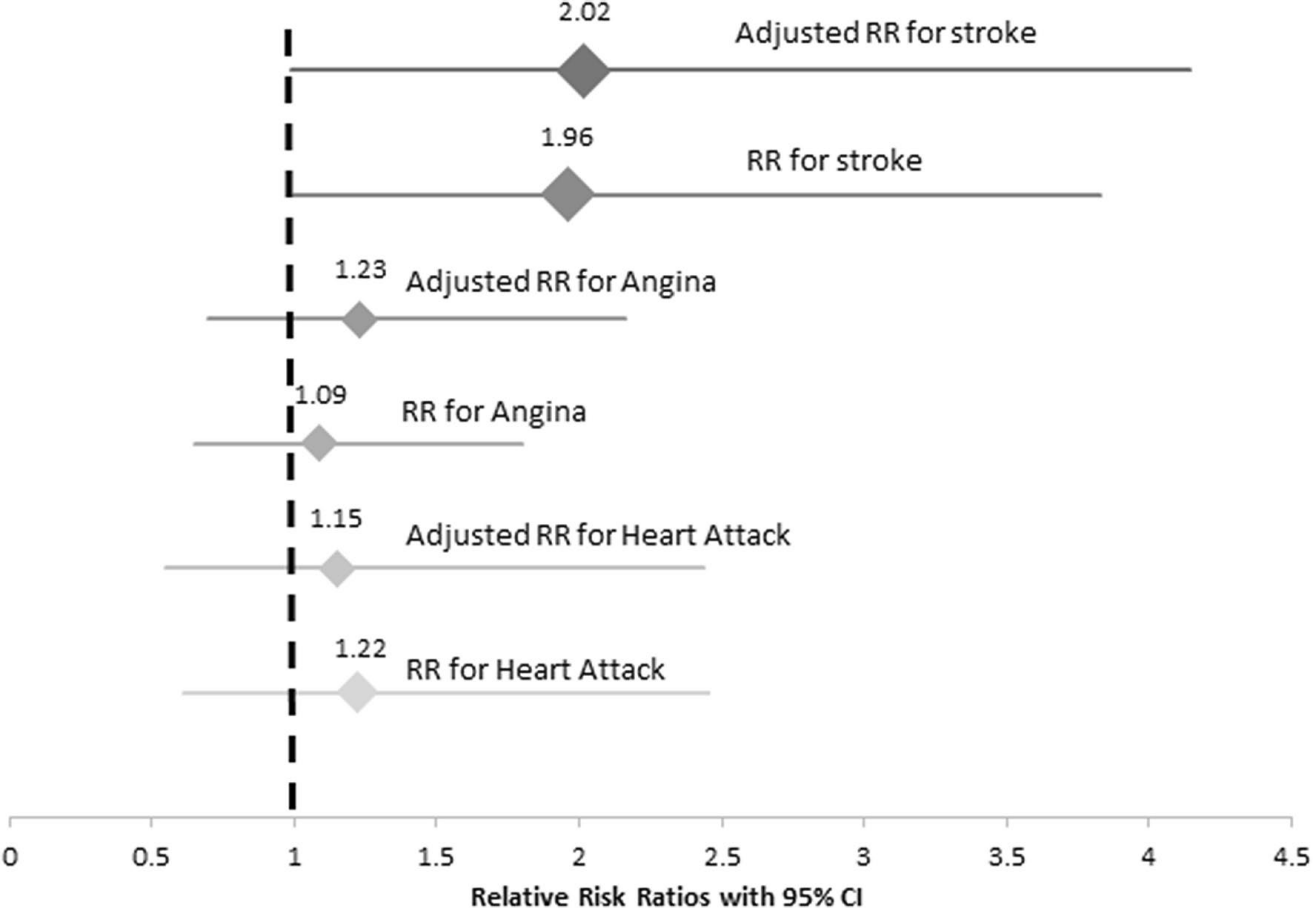
Relative Risks (RR) for CVD on exposure of Diabetes. Relative risk ratios (on the horizontal axis) for stroke, angina and heart attack are plotted along with their confidence intervals.

Estimation of prevalence of CVD (heart attack, stroke and angina) shows an increased rate of heart attack (14% vs 11.8%) and stroke (19.8% vs 10%) in DM as compared to nDM (Table 4). Next, to evaluate whether diabetes is a risk factor for developing CVD, we performed relative risk (RR) analysis. These RR were adjusted for Age and BMI with 95% confidence interval (CI) (Fig. 1). Our results indicate an increased relative risk of developing CVD in participants with diabetes for heart attack (RR: 1.22, 95% CI: [0.616, 2.45]), stroke (RR: 1.96, 95% CI: [1.00, 3.83]), and angina (RR: 1.09, 95% CI: [0.65–1.80]). The adjusted relative risk (ARR) were computed to be 1.15 with 95% CI: [0.547, 2.44], 2.02 with 95% CI: [0.98, 4.14], and 1.23 with 95% CI: [0.70, 2.16], respectively. However these results were not statistically significant (p-value > 0.05). These results are consistent with literature reports that diabetes increases the risk of developing CVD^53^. This also aligns with our aforementioned finding that reported significantly higher levels of GHB in individuals who had suffered stroke thus indicating diabetes as a risk factor of CVD (Table 3).

**Table 4:** Prevalence of the CVD amongst nDM and DM.

	nDM	DM
	(n = 119)	(n = 96)
**Heart attack**		
Number of patients	14	14
Total sample size	118	96
Prevalence (CI)	11.8% (0.0720–0.1893)	14% (0.0889–0.2300)
**Stroke**		
Number of patients	12	19
Total sample size	119	96
Prevalence (CI)	10% (0.0586–0.1680)	19.8% (0.1305–0.2886)
**Angina**		
Number of patients	25	22
Total sample size	119	96
Prevalence (CI)	21% (0.1465–0.2918)	22% (0.1565–0.3227)

The prevalence of different cardiovascular disorders including heart attack, stroke and angina amongst the American-Mexican cohort with and without diabetes. Confidence intervals for proportion have been estimated by Wilson score.

### Association between heart attack, triglycerides, TNFa, IL6 and HDL-C in those with diabetes

To identify the key determinants of heart attack amongst those with diabetes, we segregated our subset on the basis of heart attack status in diabetes (n = 96). We evaluated each parameter using Mann–Whitney U test and identified a significant association between triglycerides and heart attack amongst those with diabetes (P = 0.02*). Next, we set out to determine the underlying association between heart attack and triglycerides while controlling for TNFα, IL6 and HDL-C. For that, we converted triglycerides, TNFα, IL6 and HDL-C data to dichotomous categories, high and low. The thresholds for “*Low”* category were set at triglycerides < 200 mg/dL, TNFα ≤ 8.7 pg/mL, IL6 < 5.0 pg/ml, and HDL-C < 40 mg/dL while triglycerides > 200 mg/dL, TNFα > 8.7 pg/mL, IL6 > 5.0 pg/ml and HDL-C > 40 mg/dL were classified as *“High”*^54–57^. Chi-square statistic and Odds ratio were calculated using Cochran–Mantel–Haenszel test and results are shown in Table 5. Our results indicate that participants with diabetes and elevated levels of triglycerides were 12.68 times more likely to have a heart attack. Further, the odds of heart attack increased up to 15 times for participants with elevated levels of TNFα in presence of high triglyceride expression. Taken together, our results show that the odds of having a history of heart attack in presence of elevated triglycerides and pro-inflammatory parameters (TNFα and IL6) are approximately eight fold higher as compared to heart attack with elevation only in the levels of TNFα and IL6 (Table 5).

**Table 5:** Association between heart attack and different combinations of triglycerides, TNFa, IL6 and HDL-C, in nDM and DM.

Mantel–Haenszel common odds ratio				
Association	Estimate for DM	p-value	Estimate for nDM	p-value
Heart attack & TNFa & IL6	1.8	0.345	0.74	0.69
Heart attack & triglycerides	**12.68**	.017*	2.22	0.17
Heart attack & triglycerides & TNFa	**15.09**	0.01*	2.44	0.13
Heart attack & triglycerides & TNFa & IL6	**14.35**	0.011*	2.29	0.16
Heart attack & triglycerides & HDL-C	**15.903**	0.012*	2.169	0.191
Heart attack & triglycerides & HDL-C & TNFa	**18.8**	0.008**	2.44	0.13
Heart attack & triglycerides & TNFa & IL6 & HDL-C	**20.594**	0.007**	2.195	0.183

Shows estimates of common odds ratio for different associations between DM and nDM. Results shown in bold indicate significant findings.

*P < 0.05, **P < 0.01.

**Figure 2:**
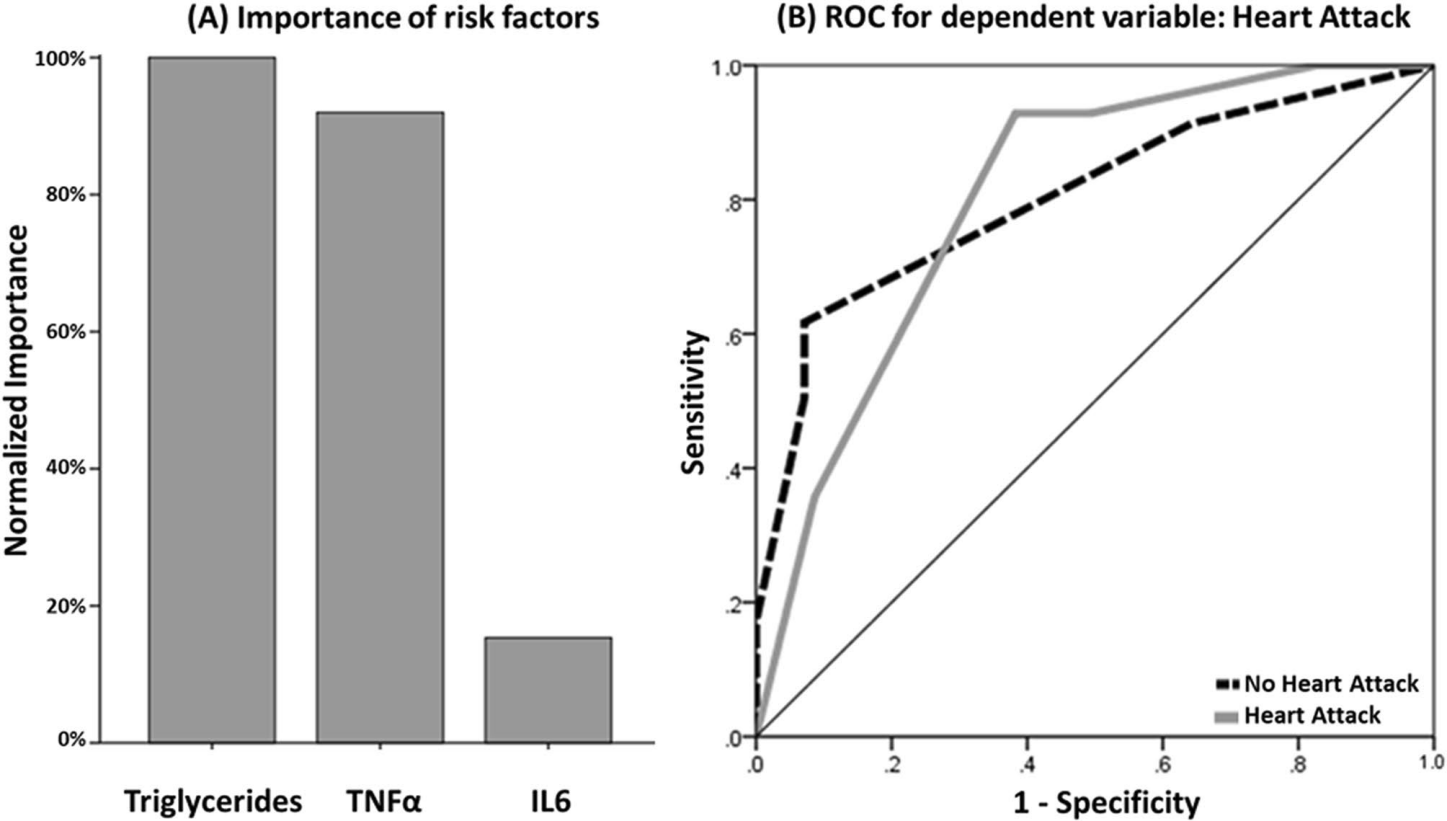
Normalized importance for three independent variables and ROC curve for dependent variable. (**A**) Showing the importance of each independent variable in predicting the occurrence of heart attack hence proving to be important risk factors in DM. (**B**) Shows the ROC curve for the predictive model of heart attack for the neural analysis. Area under the curve (AUC) of 0.803 is indicative of the accuracy of sensitivity analysis.

In order to validate these findings, we further used TNFα, IL6, and triglycerides to train a neural network for predicting heart attack. The classification table of test and training datasets for the neural network is provided in Table 6. The results indicate that from among the metabolic markers that were used in this study, triglycerides levels are the most important metabolic marker for predicting heart attack (Fig. 2-A). The ROC was constructed and area under the curve was estimated at 0.803 (Fig. 2-B).

**Table 6:** Classification table for predicting heart attack using 3 factors.

Sample	Predicted		
	No heart attack	Heart attack	Percent correct (%)
**Training**			
No heart attack	54	0	100.0
Heart attack	11	0	0.0
Overall percent	100.0%	0.0%	83.1
**Testing**			
No heart attack	27	0	100.0
Heart attack	3	0	0.0
Overall percent	100.0%	0.0%	90.0

Dependent variable is heart attack with DM + ve, predicting for three independent variables i.e. triglycerides, TNFα and IL6.

Next, we assessed the association between high levels of triglycerides, low levels of HDL-C and heart attack in those with diabetes in presence of pro-inflammatory markers (IL6, TNFα). Cochran Mantel–Haenszel test was employed to the effect. Our results shows that odds of heart attack increase by 20 fold if diabetes is associated with high levels of triglycerides, TNFα and IL6 in addition with low levels of HDL-C, (Table 5).

**Figure 3:**
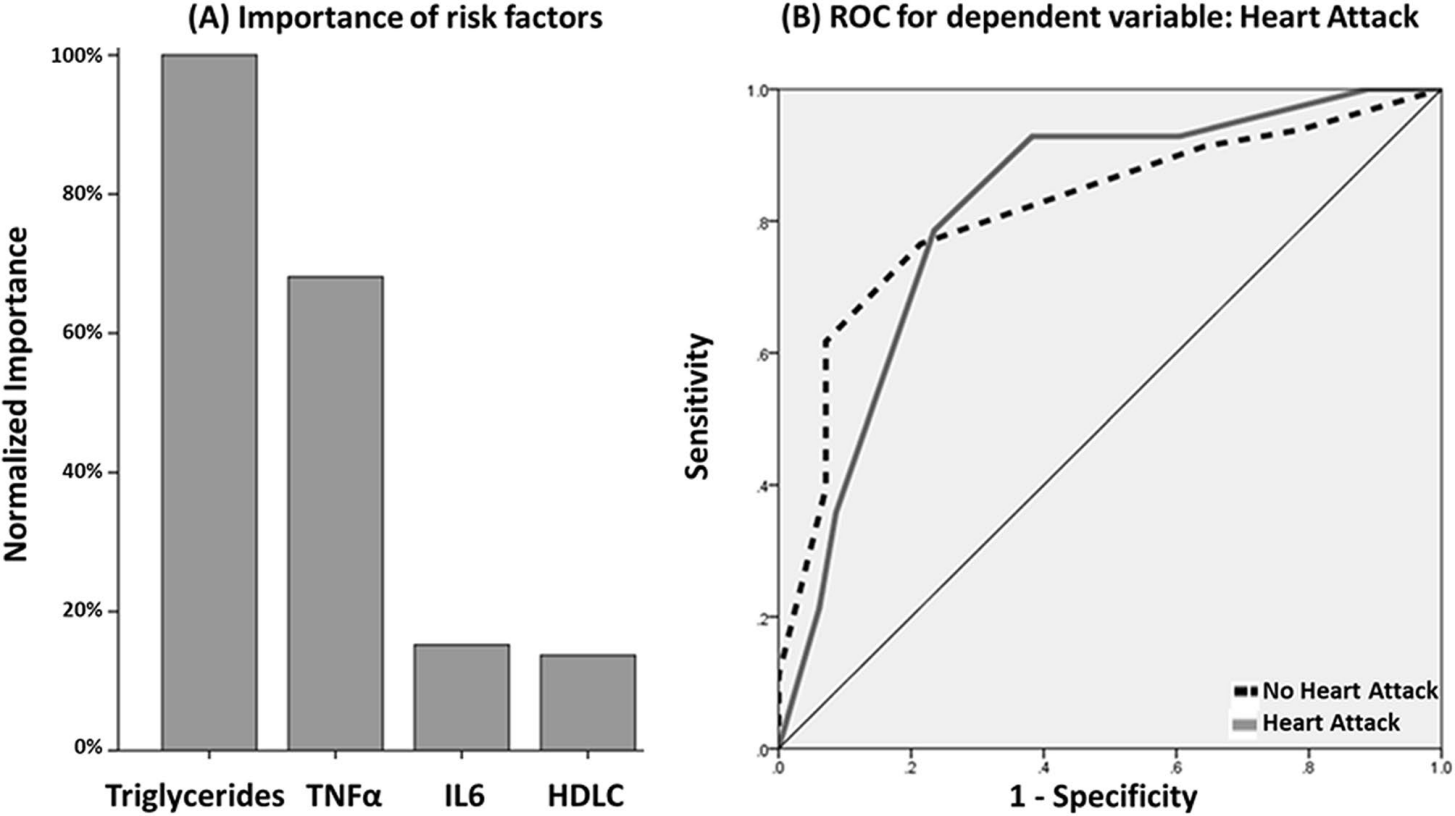
Normalized importance for four independent variables and ROC curve for dependent variable. (**A**) Showing the importance of each independent variable in predicting the occurrence of heart attack hence proving to be important risk factors among DM. (**B**) Shows the ROC curve for the predictive model of heart attack for the neural analysis. Area under the curve (AUC) of 0.817 is indicative of the accuracy of sensitivity analysis of a good model.

We validated these findings by training a neural network of four factors TNFα, IL6, and triglycerides to predict heart attack. The classification table of test and training datasets for the neural network is provided in Table 7. The results again indicated that triglycerides levels are the most important metabolic marker for predicting heart attack (Fig. 3-A). The ROC was constructed and area under the curve was estimated at 0.817 (Fig. 3-B).

**Table 7:** Classification Table for predicting heart attack for 4 factors.

Sample	Predicted		
	No heart attack	Heart attack	Percent correct (%)
**Training**			
No heart attack	52	0	100.0
Heart attack	11	0	0.0
Overall percent	100.0%	0.0%	82.5
**Testing**			
No heart attack	29	0	100.0
Heart attack	3	0	0.0
Overall percent	100.0%	0.0%	90.6

Dependent variable is heart attack with DM positive status predicting for four independent variables including triglycerides, TNFα, IL6 and HDL-C.

### Association between poorly controlled diabetes and stroke in those with diabetes

Our results revealed that GHB levels were significantly elevated in participants with a history of stroke (Table 3-B). To evaluate this association between poorly controlled diabetes and stroke, we computed odds ratio. The results show that uncontrolled diabetes increases the odds of stroke by over 3 times (Table 8). Further, to assess this relationship in presence of high levels of TNFα and IL6, we applied Cochran Mantel–Haenszel test. Consistent odds were observed for stroke while controlling for different combinations of uncontrolled diabetes, TNFα and IL6 (Table 8).

**Table 8:** Association between Stroke and poorly controlled diabetes.

Mantel–Haenszel common odds ratio		
Association	Estimate for DM	p-value
Stoke & uncontrolled diabetes	3.4	0.12
Stoke & uncontrolled diabetes & TNFα	3.34	0.11
Stoke & uncontrolled diabetes & IL6	3.27	0.12
Stoke & uncontrolled diabetes & TNFα & IL6	3.19	0.12

Shows estimates of common odds ratio for different associations among DM for stroke.

## Discussion

In this study, we have investigated the association between lipid profiles and inflammatory markers in Mexican-Americans with diabetes towards estimating the odds of having CVD. Although hyperlipidemia can be classified as major risk factor for CVD but different ethnicities can exhibit disparities in its prevalence. According to previous cross-sectional and retrospective studies conducted on Asian Americans, Mexican Americans and Blacks, distinct patterns of dyslipidemia have been reported^58,59^. Hence, risk factors in addition to ethnic/racial minority-specific single nucleotide polymorphism (SNPs) can help in better understanding and prevention of CVD risk and associated mortality^60^. Our results from the current study show that diabetes is a major contributor to inflammatory changes in Mexican-Americans, which in addition to the dyslipidemia can culminate in CVD outcomes.

### Pro-inflammatory cytokine profile is associated with diabetes

Insulin resistance underlying type-2 diabetes (T2DM) superimposed on obesity is known to elevate plasma levels of inflammatory markers such as IL-6, TNFα. Two different underlying mechanisms have been proposed for this inflammatory response. Firstly, high glucose intake and over-nutrition can lead to oxidative stress besides inducing an imbalanced inflammatory response^30^. Secondly, higher levels of inflammatory mediators (IL-6 and TNFα) lead to disruption of insulin action due to suppression of insulin transduction^61^. The results from our study are consistent with these literature reports as a significant elevation was observed in inflammatory markers amongst the DM (Table 1). Further, data from the European nested case–control prospective study showed no change in IL-1β between the DM and non-disease participants besides higher levels of IL-6 and TNFα^62^. This finding is also in agreement with our pro-inflammatory profile analysis (Table 1).

### Relative risk of CVD amongst DM

Amongst all the risk factors associated with CVD, diabetes contributes significantly towards CVD related mortalities^63^. A prospective study from the Framingham cohort also concluded higher incidence of CVD in men and women with diabetes^8^. Our results exhibit a similar trend with a higher prevalence of heart attack and stroke in DM as compared to nDM (Fig. 1).

### Uncontrolled glucose and CVD

Diabetes leads to changes at micro- and macro-vascular levels that culminate into a variety of clinical complications. Specifically, diabetes increases the susceptibility of an individual to cerebral small and large vessel diseases^64,65^. Thus, increased glucose levels (hyperglycemia) lead to elevated risk for outcomes such as ischemic stroke etc. Our study revealed that the relative risk for stroke was highest amongst individuals with diabetes after adjusting for age and BMI (Fig. 1). In an earlier multiethnic prospective study, the relationship between fasting blood glucose and ischemic stroke was evaluated along with the importance of how strict glucose control can prevent stroke^66^. These findings are coherent with our study, where a significant increase in HbA1c was observed amongst participants with a history of stroke (Table 3B). Moreover, the odds of stroke were observed to increase by up to 3 times in patients with poorly controlled diabetes (Table 8) further supporting the association between stroke and poorly controlled diabetes.

### Triglycerides and CVD

Elevated levels of triglyceride are known to cause impairment in lipoprotein metabolism which leads to an increase in the risk of CVD^67,68^. Our statistical analysis shows that triglyceride levels are significantly higher in DM thus linking them closely to T2DM (Table 1). High levels of triglycerides are considered as an epiphenomenon of the metabolic syndrome and insulin resistance^68,69^, which can help identify CVD susceptible candidates with low levels of HDL-C and small LDL particles^70^. Furthermore, analysis performed by National Health and Nutrition Examination Survey (NHANES) showed strongest association of triglycerides with risk of CVD in comparison to other components of metabolic syndrome^71^. Here, we showed that participants with a history of heart attack patients exhibit a significant increase in triglyceride expressions (Table 3-A). Further, our risk analysis shows that the odds of heart attack increases by up to 5.7 fold in DM as compared nDM (Table 5).

### HDL-C and CVD

Benitez et al. reported the relationship between HDL-C and CVD all-cause mortality^72^. Prospective and epidemiological investigations also show an inverse association between HDL-C and CVD with the risk of coronary heart disease (CHD) lowered by 2–3 percent for every 1 mg/dl increase in HDL-C^73^. In the current study, this association between high levels of triglycerides, while controlling for the low levels of HDL-C, showed that the odds of heart attack increase by 7.36 times in DM as compared to nDM (Table 5).

### Association of lipid profile and inflammatory markers with CVD

Elevated triglycerides and lowered HDL-C levels in serum are common metabolic abnormalities associated with insulin resistance^68,69^. Hence, hypertriglyceridemia can act as an indicator of the metabolic syndrome and type-2 DM. Further, the ratio of triglyceride levels to HDL-C can be employed as a clinical marker for insulin resistance which can then be a surrogate for measuring the impact of diabetes on the CVD incidence^70^. Importantly, previous prospective studies on triglycerides have also indicated a strong association between CVD risk and lowered levels of HDL-C, LDL-C in individuals with T2DM^71^. The current study evaluates different combinations of lipid profile (high triglycerides and low HDL-C levels) and increased levels of the hyperglycemia-induced inflammatory mediators in increasing the odds of heart attack (Table 5). Moreover, the proposed neural network model of these major risk factors can assist in accurately predicting heart attacks in DM (Figs. 2 and 3).

### Ethics approval and consent to participate

All randomly recruited participants were informed and provided a written consent. Institutional review board (IRB) approval was obtained before conducting the study by The Committee for the Protection of Human Subjects Office of Research Support Committees, UT-Houston—SPH—Brownsville Regional Camp/RAHC (Reference No. HSC-SPH-09-0276).

### Consent for publication

Consent was provided by all authors.

## Conclusion

This study concludes that diabetes is associated with low-grade inflammation and that raised levels of triglycerides and low levels of HDL-C in DM increases the risk of cardiovascular disease (CVD) outcomes. Prevalence analysis of CVD in diabetes mellitus (DM) in comparison to nDM is indicative of DM as a major risk factor in CVD. Moreover, elevation of the inflammatory markers resulting from DM with concomitant increase in the lipid profile amongst DM contributes to heart attack.

## Data Availability

The datasets used and/or analyzed during the current study are available from the authors on reasonable request.

## Abbreviations

CVD: Cardiovascular disorders
CCHC: Cameron County Hispanic Cohort
FBG: Fasting blood glucose
ADA: American Diabetes Association
TNFα: Tumor necrosis factor alpha
IL6: Interleukin-6
HDL-C: High density lipid cholesterol
LMIC: Low and middle income countries
WHO: World health organization
NHANES: American National Health and Nutrition Examination Survey
HF: Heart failure
AHA: American Heart Association
JNC: Seventh Joint National Committee
BMI: Body mass index
CRP: C-reactive protein
RAGE: Receptor for advanced glycation end
IBM: International Business Machine
DM: Diabetes
nDM: Without diabetes
RR: Relative risk
OR: Odds ratio
CI: Confidence interval
T2DM: Type-2 diabetes
