# Author Correction: An evaluation of lipid profile and pro-inflammatory cytokines as determinants of cardiovascular disease in those with diabetes: a study on a Mexican American cohort

**DOI:** 10.1038/s41598-021-93445-9

**Published:** 2021-07-05

**Authors:** Amna Tahir, Perla J. Martinez, Fayyaz Ahmad, Susan P. Fisher-Hoch, Joseph McCormick, Jennifer L. Gay, Shaper Mirza, Safee Ullah Chaudhary

**Affiliations:** 1 Biomedical Informatics Research Laboratory, Department of Biology, Lahore University of Management Sciences, Lahore, Pakistan; 2 Department of Epidemiology, Human Genetics and Environmental Health, School of Public Health, University of Texas Health Science Center – Brownsville Regional Campus, Brownsville, USA; 3 Department of Statistics, University of Gujrat, Gujrat, Pakistan; 4 Department of Health Promotion and Behavior, College of Public Health, University of Georgia, Athens, USA; 5 Microbiology and Immunology Laboratory, Department of Biology, Lahore University of Management Sciences, Lahore, Pakistan

Correction to: *Scientific Reports* https://doi.org/10.1038/s41598-021-81730-6, published online 28 January 2021

Joseph McCormick was omitted from the author list in the original version of this Article.

The Author Contributions section now reads:

“S.U. and S.M. supervised the study. P.M., S.F., J.M., J.G. and S.M. developed the cohort and obtained the data. A.T., F.A., S.M. and S.U. designed the data analysis. A.T. and S.U. performed the analysis and wrote the manuscript.”

The original Article has been corrected.

